# Label-free enrichment of rare unconventional circulating neoplastic cells using a microfluidic dielectrophoretic sorting device

**DOI:** 10.1038/s42003-021-02651-8

**Published:** 2021-09-24

**Authors:** Jose Montoya Mira, Ajay A. Sapre, Brett S. Walker, Jesus Bueno Alvarez, Kyle T. Gustafson, Eugene Tu, Jared M. Fischer, Melissa H. Wong, Sadik Esener, Yu-Jui Chiu

**Affiliations:** 1 Knight Cancer Early Detection Advanced Research Center, Oregon Health & Science University, Portland, OR 97201 USA; 2 Department of Biomedical Engineering, Oregon Health & Science University, Portland, OR 97201 USA; 3 Department of Surgery, Oregon Health & Science University, Portland, OR 97239 USA; 4 The Knight Cancer Institute, Oregon Health & Science University, Portland, OR 97201 USA; 5 Department of Molecular and Medical Genetics, Oregon Health & Science University, Portland, OR 97239 USA; 6 Department of Cell, Developmental & Cancer Biology, Oregon Health & Science University, Portland, OR 97201 USA

**Keywords:** Assay systems, Isolation, separation and purification, Tumour biomarkers

## Abstract

Cellular circulating biomarkers from the primary tumor such as circulating tumor cells (CTCs) and circulating hybrid cells (CHCs) have been described to harbor tumor-like phenotype and genotype. CHCs are present in higher numbers than CTCs supporting their translational potential. Methods for isolation of CHCs do not exist and are restricted to low-throughput, time consuming, and biased methodologies. We report the development of a label-free dielectrophoretic microfluidic platform facilitating enrichment of CHCs in a high-throughput and rapid fashion by depleting healthy peripheral blood mononuclear cells (PBMCs). We demonstrated up to 96.5% depletion of PBMCs resulting in 18.6-fold enrichment of cancer cells. In PBMCs from pancreatic adenocarcinoma patients, the platform enriched neoplastic cells identified by their *KRAS* mutant status using droplet digital PCR with one hour of processing. Enrichment was achieved in 75% of the clinical samples analyzed, establishing this approach as a promising way to non-invasively analyze tumor cells from patients.

## Abstract (web summary)

Montoya Mira & Sapre et al. design a microfluidic device utilizing dielectrophoresis for the enrichment of circulating neoplastic cells that possess both epithelial and immune-like characteristics. They optimized the device using in vitro models and then applied it to clinical samples for clinically relevant mutation analyses.

## Introduction

Cancer is a major worldwide public health burden and the second leading cause of death in the United States^1^. Advances in medical technologies have led to significant progress in both treatment and diagnosis, yet clinicians rely on invasive and often risky biopsies for cancer detection. To address these limitations, the field of liquid biopsy has rapidly emerged as a non-invasive method to obtain tumor-derived biomarkers for diagnosis, staging, treatment, and prognosis^2,3^.

Circulating neoplastic cells disseminated from solid tumors are valuable biomarkers reflecting the disease transcriptome, genome, and proteome. Circulating tumor cells (CTCs) are cells disseminated from the tumor, shed into peripheral blood, and capable of recapitulating tumorigenesis. In epithelial-derived tumors, CTCs are phenotypically defined as large cells (30 µm on average) lacking expression of the pan-leukocyte marker, CD45, and expressing cytokeratin (CK). In addition, other markers have been associated with CTCs like epithelial cell adhesion molecule (EpCAM) or E-cadherin (ECAD) for isolation or identification^4^. CTC enrichment and isolation technologies exist, including the FDA-approved CellSearch^®^ technology, to enumerate and phenotype CTCs to guide clinical prognosis^5^. With estimates of one CTC per 10^6^–10^8^ leukocytes found in peripheral blood of pancreatic ductal adenocarcinoma (PDAC) patients, levels of these rare biomarkers challenge their functional utility, thus improved approaches to capturing these rare cells are needed^6,7^. Many groups have focused on using microfluidic sorting, density-based devices, size exclusion filtration, or marker specific isolation methods^8–10^. These technologies rely on either a single physical property (size, density) or on specific protein expression (EpCAM, ECAD, CK) to differentiate CTCs from peripheral blood mononuclear cells (PBMCs). However, the rarity of CTCs has greatly limited their application in providing multi-omics information that encompasses tumor heterogeneity and has not translated well into widespread clinical use.

In the past 5 years, other promising types of unconventional circulating neoplastic cells have been described such as tumor hybrid cells (THC) and circulating hybrid cells (CHCs)^11,12^, CHCs are described as having hybrid phenotypes of a neoplastic cell and a leukocyte with dual expression of CD45 and tumor markers such as EpCAM or CK^11–15^. In PDAC patients, CHCs have shown better prognostic value than CTCs as their enumeration correlated with stage and survival^12^. While CHCs are more abundant, their physical and phenotypic similarity to PBMCs makes their isolation and analysis a challenge^14^.

To address these challenges, we developed a microfluidic device utilizing the intrinsic dielectrophoretic (DEP) properties of cells to enable their label-free enrichment, in a fashion compatible with both phenotypic and genotypic downstream analyses. Particle movement caused by difference of electrical polarizability between particles and medium in a non-uniform electric field has been thoroughly elaborated by Jones^16^ and Pohl^17^, and has been applied to separate and isolate biomarkers^18–21^. When cells are exposed to this electric field, differential polarization is created between the cells and the surrounding media translating into a resultant force. This resultant force can either be positive DEP where cells travel up the electric field (E-field) gradient, or negative DEP where cells travel down the gradient^22^. The overall magnitude of the DEP force is dictated by the E-field gradient and the particle/cell geometry and the directionality of the force is determined by relative permittivity between the cell and the surrounding media and is specifically explained by the Clausius–Mossotti factor^23^.

DEP as a cell differentiation approach can be grouped into two main categories, batch mode or continuous mode processing. Batch mode processing relies on a unique frequency cut-off where a cell’s DEP response changes between positive and negative, for each discrete cell type. Defined frequencies can then be used to separate two cell groups to positive or negative DEP regimes followed by flushing out the negative DEP partition. This approach provides enhanced specificity for discrete cell types expressing narrow dielectrophoretic variance; however, its utility is impeded by low throughput and narrow search space^24–29^. In contrast, continuous mode processing uses a secondary force (flow, acoustic, or others) to incrementally bias cell trajectory over a period of time^30–32^. The combination of continuous flow and DEP can result in multiple cell sorting outcomes in high cell density environments translating into a versatile high-throughput methodology for mixed cell populations with variant dielectrophoretic responses. The majority of strategies currently employed for rare cell enrichment are reliant on clearly defined physical properties and minimal phenotypic variation of the target cell population. However, this approach fails for heterogeneous cell populations with high phenotypic and DEP variance making it unlikely for a single condition to be successful. To address these limitations, we designed and optimized a continuous mode DEP based microfluidic device to allow for enrichment of highly heterogenous rare cell populations like CHCs. The workflow was validated using beads, optimized on cell lines and  healthy PBMCs, then tested on cancer patient samples. In this work, PBMCs from a small amount of blood (2 mL) were sorted in a high-density environment where non-target immune cells were depleted from the sample enriching for neoplastic cells in a label-free manner. As a proof of concept, enriched CHCs were subjected to phenotypic and genotypic downstream analysis demonstrating clinically relevant *KRAS* mutation status in PDAC tumors.

## Results and discussion

### Design and validation strategy of microfluidic DEP sorting device

#### Design

**Fig. 1:**
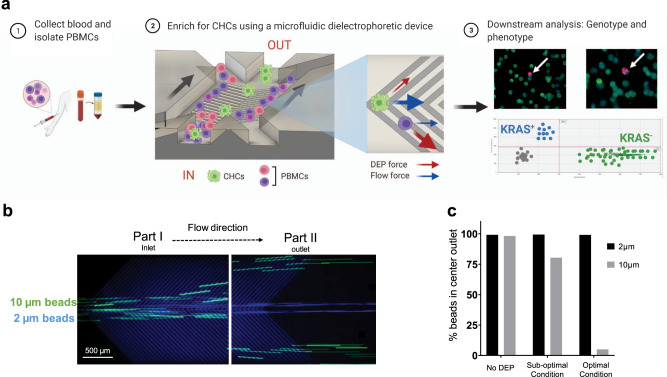
Workflow for label-free enrichment of unconventional circulating neoplastic cells and proof of concept demonstrating target bead enrichment. **a** Schematic representation of CHC enrichment from patient blood. (1) PBMCs are isolated from 2 ml of whole blood. (2) DEP microfluidic device is used to enrich for CHCs and samples is collected from outlets. (3) Downstream analysis, both phenotypic and genotypic, can be performed to obtain clinically relevant information. Created with BioRender.com. **b** Fluorescent images of microfluidic channel showing differential response of polystyrene beads, 10 µm (green) and 2 µm (blue). Part I represents the inlet and part II represent the outlet. Scale bar is 500 µm. **c** Percent of beads collected from the center outlet at an optimal condition (5 MHz), a sub-optimal condition (300 kHz), and with no applied DEP.

The concept of enriching a target cell population by depleting non-relevant cells is shown schematically in Fig. 1. First, the sample is infused into the microfluidic DEP sorting device, composed of a microfluidic channel and interdigitated electrode arrays. The microfluidic device was manufactured by standard photolithographic and lift-off techniques where a silicon substrate was patterned with an interdigitated electrode layer and a polydimethylsiloxane (PDMS) layer was bonded on top to form a fluidic channel. To allow cells throughout the channel’s height to be acted upon by DEP forces, the chamber height was designed to be 25 µm or approximately twice the average size of PBMCs. Under continuous flow the sample is centrally confined by two sheath flows providing a small variation of initial speed and position. The interdigitated electrodes were designed to have a width and spacing of 35 µm, approximately 2–3 times the size of PBMCs, to minimize cells being captured on the electrodes by DEP forces (Supplementary Fig. 1). The V-shape electrode geometry was designed to deflect PBMCs in a high cell density environment by utilizing DEP forces to deflect cells along the electrodes while flow forces direct cells along the length of the device^33–35^. Cells experiencing strong DEP forces are depleted to the side outlets, while less responsive cells are enriched in the center channel (Fig. 1a). In order to maximize the DEP response and minimize cells trapped on electrodes, a conductive buffer at 145 mS/m (approximately 0.1x PBS) was used during cell enrichment processing for both the sample and sheath inlets. The buffer was chosen to balance cell viability, DEP reactivity, and electrode corrosion^31,36^. For ease of use and rapid troubleshooting the complete setup was placed under a microscope for continuous viewing where the fluidic channel is visible under a standard 5x objective lens. This setup allows for sample compatibility with genotypic or phenotypic downstream analysis (Fig. 1a) which can be helpful for understanding cellular clinical significance^37^.

#### Validation

To demonstrate the concept of enriching by depleting a target population we used a sample mix composed of 2 µm (blue) and 10 µm (green) fluorescent polystyrene beads of identical chemistry (Fig. 1b, c). The sample mix was introduced to the inlet of the microfluidic DEP sorting device with AC voltage applied to the interdigitated electrodes, and particle movement was monitored by live imaging. We defined the optimal condition as the parameters where one type of particle was enriched by depletion of the other particle. To find this condition, the frequency was swept from 0.1 MHz to 10 MHz at 0.1 MHz increments at constant voltage. Particles were collected at the outlets and analyzed by flow cytometry.

Under the optimal frequency, both bead populations were closely aligned with the center of the channel while near the sample inlet (Fig. 1b, part I), however only the 10 µm beads were displaced further downstream by DEP forces revealing particle trajectory bias over time and space (Fig. 1b, part II). Under this condition, 95% of the 10 µm beads were depleted from the center due to the application of strong positive DEP forces, while less than 1% of the 2 µm beads were depleted to the sides (Fig. 1c). Figure 1c also shows an example at one of the sub-optimal frequencies that only 19.5% of 10 µm were depleted, suggesting that the magnitude of the DEP force was smaller than the dominant flow forces. To confirm that DEP forces were the primary driver of bead depletion, the device was run without applied DEP and resulted in 99% of both bead populations remaining in the center unaffected by drift. This workflow demonstrated that depletion of one population by DEP can result in the collection of targets with high purity under proper working parameters. To understand how this applied to a complex biological sample mix, we next tested this process on PBMCs and cell lines.

### Optimization of PBMC depletion and hybrid cell line enrichment

#### PBMC DEP response characterization

**Fig. 2:**
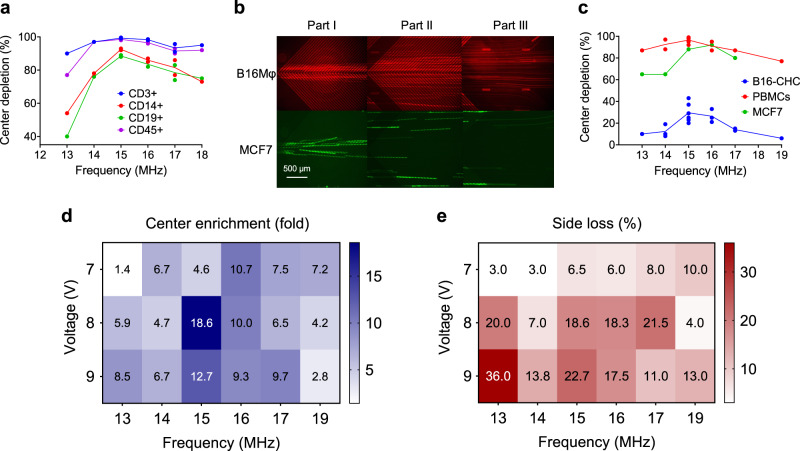
DEP response characterization of PBMCs from healthy donors and cancer cell lines. **a** PBMC subpopulation DEP respsone as presented by depletion of CD3^+^ (blue line), CD14^+^ (red line), CD19^+^ (green line), and CD45^+^ (purple line) cells across frequencies, 13 MHz to 18 MHz at a constant 8 Vpp by flow cytometry. **b** Fluorescent images near the inlet (Part I), at the center between batches of electrodes (Part II), and close to the outlet (Part III) of the microfluidic channel revealed depletion of MCF7 (green) and less reactive B16Mϕ (red) at 15 MHz and 8 Vpp. Scale bar is 500 µm. **c** Percent depletion of B16Mϕ, MCF7, and PBMCs from the center outlet across frequencies, 13 MHz to 19 MHz, at a constant 8 Vpp by flow cytometry. **d** Center enrichment heatmap with voltage (V) in the y axis and frequency (MHz) in the *x* axes (*n* = 1–5, independent experiments). **e** Percentage side loss heatmap with voltage (V) in the y axis and frequency (MHz) in the *x* axes (*n* = 1–5, independent experiments).

As DEP conditions can be optimized to separate discrete physiologic objects, we set out to establish the optimal condition where hybrid cells could be enriched from PBMCs. Cells are biologic entities, thus their conductivity, permittivity, and physiological state are the main drivers of differential DEP response^38^. Healthy PBMCs are composed of heterogeneous populations, but tend to be relatively similar in nature across individuals, therefore healthy PBMCs act as an ideal object to be optimized for depletion. PBMCs are mainly comprised of lymphocytes and monocytes. Of these, T-cells represent the majority (70–80%) of the lymphocytes, therefore we hypothesized that of all healthy PBMCs depleting T-cells would be the primary contributor of neoplastic cell enrichment. First, to understand the DEP response of PBMCs as a collective of cells and as individual subpopulations, we conducted the workflow using PBMCs isolated from whole blood (*n* = 5) from healthy subjects. PBMCs were diluted in DEP buffer (1 × 10^7^ cells/mL), and 1 × 10^6^ cells were loaded into the central inlet for each parameter, then subjected to device processing for at least 30 min. Cells were not responsive to DEP at low frequencies, thus we focused analyses when cells were visually responsive, between 13 and 17 MHz at a constant voltage of 8 Vpp. We selected this voltage because cells were minimally reactive below 7 Vpp but were trapped on the electrodes at voltages greater than 9 Vpp. For each frequency, cells were collected from all three outlets, then stained with antibodies against CD45 (pan-leukocyte), CD3 (T-cell receptor), CD14 (macrophage), and CD19 (pan B-cell) (Fig. 2a). Depletion of each cell type was calculated by flow cytometry analysis of the outlets where the percent of CD3^+^, CD14^+^, or CD19^+^ cells in the side channels was divided by the percent of the cell of interest from the total cells processed (CD45^+^). A similar trend was observed for all populations, where 15 MHz resulted in maximum depletion from the center channel, which was most pronounced for CD3^+^ cells (99.3%), followed by CD14^+^ cells (92.5%), and CD19^+^ cells (88.5%). Ultimately, only 1.5% of the initial number of PBMCs (CD45^+^) were collected in the central outlet (Fig. 2a). Of the subpopulations, we found CD3^+^ T-cells were the most responsive and had similar depletion levels between 14, 15, and 16 MHz at 97%, 99.3%, and 98.3%, respectively. In contrast, depletion of CD19^+^ and CD14^+^ cells significantly changed from 13 MHz to 15 MHz, suggesting that these cell populations were more sensitive to specific DEP parameters. We corroborated the previous findings by Gascoyne et al. that different cell types have different DEP response profiles^39–41^. Not only did we observe PBMCs depletion, we found that the PBMCs composition changed post-depletion e.g. there was 7.3-fold and 4.6-fold enrichment of CD19^+^ and CD14^+^ cells in the center channel, respectively. These results suggest that across PBMC phenotypes there is a cell type-dependent DEP response that supports their differential separation and that depletion of CD3^+^ cells could drive enrichment of rare cells.

#### In vitro CHC DEP response characterization

Next, to understand the DEP response of malignant cells we selected an established in vitro hybrid cell line which was studied in depth previously by Gast et al. B16Mϕ–RFP, generated through the fusion of B16F10 and bone marrow-derived macrophages as a model of CHCs^12^. In addition, a breast cancer cell line, MCF7, was used as a surrogate of traditional CTCs. Previously, Gast et al. characterized the phenotypic hybrid nature of in vitro derived CHCs. Conveniently these hybrids co-expressed nuclear RFP and cytoplasmic YFP facilitating their differentiation from the MCF7-GFP cells by flow cytometry^12^. To optimize conditions for hybrid cell enrichment, cancer cells were diluted in DEP buffer at 1 × 10^7^/mL and only 1 × 10^6^ cells were loaded into the microfluidic DEP sorting device for each parameter. To visualize cancer cell DEP response, fluorescent images were taken at three locations (Fig. 2b), near the inlet (part I), the middle after the first batch of electrodes (part II), and close to the outlets (part III). At 15 MHz/8 Vpp, both B16Mϕ−RFP and MCF7-GFP were aligned with the central inlet flow. As the cells flowed through the device, the majority of MCF7 cells depleted to the sides while the majority of B16Mϕ remained in the center. To quantify each sorting parameter, we processed cells in the device for at least 30 min and analyzed all the outlets by flow cytometry. Across all tested frequencies MCF7 cells were more responsive than B16Mϕ cells. MCF7 cells had a minimum response at 13 MHz where 65% of the cells were depleted and a maximum response at 16 MHz where 92% were depleted to the sides (Fig. 2c). In contrast, the minimum DEP response for B16Mϕ was at 19 MHz where only 6% of the cells were depleted and the maximum DEP response was at 15 MHz where only 29.5% of cells were depleted suggesting that hybrid cells have a unique non-responsive DEP profile (Fig. 2c). In parallel, we analyzed the DEP response of healthy PBMCs (CD45^+^) under the same conditions. Again, PBMCs were highly responsive across all frequencies with a minimum response at 19 MHz with 77% of the cells depleted and a maximum response at 15 MHz with 96.5% of the cells depleted from the center channel (Fig. 2c).

Overall, we found a larger differential response between B16Mϕ-RFP and PBMCs as compared to MCF7-GFP cells and PBMCs despite their close proximity in size to PBMCs suggesting that label-free size-independent enrichment was possible within our workflow. This also suggests that B16Mϕ-RFP cells inherently have different dielectric properties likely resulting from their hybrid nature. Although numerous studies focus on CTC enrichment and isolation, these strategies rely on either the inherent large size of CTCs or specific membrane protein expression. CHCs are a promising alternative as they are more abundant than CTCs, however there is a lack of technologies focused on their enrichment, a critical factor in developing CHCs as a cancer biomarker. This data highlights the potential of using DEP to enrich for CHCs and potentially other rare cellular biomarkers that are difficult to differentiate phenotypically.

### Hybrid cell line spiked into healthy peripheral blood samples

A number of technologies focused on rare cell analysis rely on the isolation of single cells, which is challenging. Recently, the development of high-throughput single-cell technologies allows for the analysis of thousands of cells and demonstrates the need for rare cell enrichment rather than pure isolation. For most enrichment strategies there is a tradeoff between total enrichment and overall cellular biomarker loss during processing due to a limit of detection (LOD) threshold. To find the optimal frequency and voltage parameters to enrich for CHCs, we evaluated the enrichment of B16Mϕ-RFP cells from PBMCs. PBMCs isolated from healthy subjects were spiked with 50,000 B16Mϕ cells (50,000 per 1 × 10^6^ PBMCs), then subjected to DEP for at least 30 min, and analyzed for cells types isolated from all outlets by flow cytometry. Enrichment of hybrids in the center channel and depletion in the side channels was calculated using flow cytometric data for each outlet (see Eq. 1 and Eq. 2). Unlike other strategies for rare cell isolation focused on the absolute number of recovered rare cells, our strategy utilized the ratio of rare cells to total PBMCs as the rating of enrichment with the goal of enriching samples above the LOD for downstream analytics. This process was repeated for six frequencies, from 13–18 MHz and three voltages for each frequency, 7–9 Vpp. A heatmap was generated to assess center channel hybrid enrichment (fold change) and side channel hybrid loss (percentage loss) taking into account both the frequency and voltage applied to the device (Fig. 2d, e). The enrichment varied from 1.4-fold and 2.8-fold at 13 MHz/7 Vpp and 19 MHz/9 Vpp, respectively, to 18.6-fold and 12.7-fold at 15 MHz/8 Vpp and 15 MHz/9 Vpp, respectively. The highest B16Mϕ enrichment identified was 18.6-fold (*n* = 5) at 15 MHz/8 Vpp (Fig. 2d). The voltage was a strong driver of the magnitude of DEP response with greater loss of B16Mϕ cells to the sides at higher voltages (Fig. 2e). For example, at 15 MHz, there was 6.5% loss to the sides at 7 Vpp, 18.6% at 8 Vpp, and 22.7% at 9 Vpp. Overall, we found that 15 MHz and 8 Vpp provided optimal DEP parameters for B16Mϕ hybrid enrichment and potentially for patient-derived CHCs. These trends observed follow previously describe bell-shaped DEP response curves^42^. In these models as well as in the data presented here, a narrow range of maximum DEP response can be appreciated. Overall, our unique strategy to deplete healthy PBMCs from the sample drives enrichment of the target tumor cell population.

1 $${{Center}}\,{{Enrichment}}=\frac{\left(\frac{\#\,{{of}}\,B16M\phi \,{{in}}\,{{center}}\,{{channel}}\,{{after}}\,{{sorting}}}{\#\,{{of}}\,{{PBMCs}}\,{{in}}\,{{center}}\,{{channel}}\,{{after}}\,{{sorting}}}\right)}{\left(\frac{\#\,{{of}}\,B16M\phi \,{{total}}}{\#\,{{of}}\,{{PBMCs}}\,{{total}}}\right)}$$

2 $${{Side}}\,{{Loss}}=\frac{\#\,{{of}}\,B16M\phi \,{{in}}\,{{side}}\,{{channels}}\,{{after}}\,{{sorting}}}{\#\,{{of}}\,B16M\phi \,{{total}}}$$

According to current literature there are approximately 30 CHCs per 500,000 PBMCs in PDAC patients representing only 0.006% of total cells in blood^13^. This overall rarity highlights a clear need for CHC enrichment for downstream analyses such as mutational profiling using ddPCR or genomic surveying using single-cell sequencing, both of which have a limit of detection (LOD) of 0.1%^43–46^. With 18.6-fold enrichment using the described workflow, the microfluidic DEP sorting device described could enrich above 0.1% LOD suggesting there is utility targeting the depletion of healthy PBMCs to facilitate a more comprehensive understanding of poorly understood circulating neoplastic cells.

Finally, since a strong electric field can induce cell death, which would disrupt downstream analyses, we assessed cell viability throughout the DEP workflow. Non-viable cells can lead to aggregation and clogging, which reduces device performance and sensitivity for isolating cellular biomarkers. The low conductive DEP buffer was designed to maintain cell viability while allowing for DEP separation. In order to study cell viability, the effect of DEP buffer alone and under applied DEP was tested. A549 cells were placed in DEP buffer and cell viability was assessed over time using propidium iodide staining via flow cytometry. A549 cells had 98% viability after 30 min in DEP buffer and 97% after 120 min (Supplementary Fig. 2). To measure cell viability post-DEP processing at 15 MHz/8 Vpp, PBMCs were stained with calcien AM from the outlets and immediately measured via flow cytometry (Supplementary Fig. 2). The side outlets had 99% viability while the center outlet had 97% viability, suggesting that enrichment via DEP is compatible with analytic techniques that require cell viability, morphology, and nucleic acid integrity to be maintained.

### Label-free Enrichment of *KRAS* mutant cells from PDAC patient samples

**Fig. 3:**
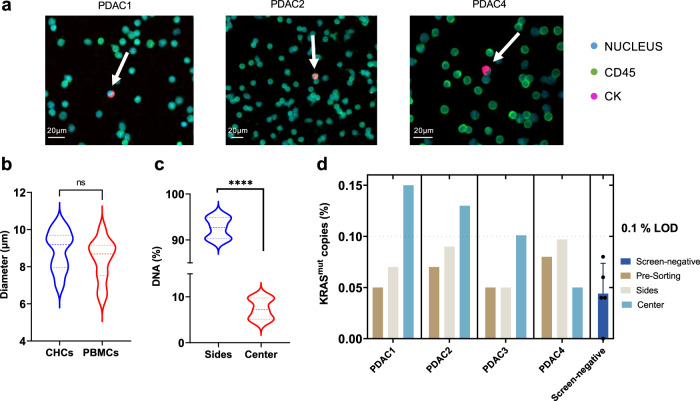
Downstream analysis of clinical PDAC samples processed with the DEP device. **a** In situ immunofluorescence microscopy analysis of PBMCs before device processing, arrows indicate presence of CHCs (Hoescht^+^/CD45^+^/CK^+^). Scale bar is 20 µm. **b** Average diameter (µm) of CHCs and PBMCs from in situ immunofluorescence images. **c** Average clinical sample DNA extracted from cells collected from all outlets (*n* = 4, independent experiments). **d** Percent *KRAS*^mut^ copies for each patient (*n* = 4, independent experiments) from all outlets compared to average *KRAS*^mut^ copies for screen-negative controls (*n* = 5, independent experiments) measured by ddPCR.

To evaluate the ability of the microfluidic DEP sorting device to provide relevant clinical information when assessing peripheral blood from cancer patients, we set out to demonstrate that DEP-enriched CHCs could be used to evaluate *KRAS* mutational status. Whole blood from four patients with PDAC (stage III-IV) was collected. All patients had clinically confirmed *KRAS*^mut^ primary tumors (Table 1). The majority of PDAC tumors harbor *KRAS* mutations and its presence in circulation, from circulating cells or cell-free DNA, is clinically relevant for cancer detection^47^. Moreover, our group has previously found that a subpopulation of FACS isolated CHCs have *KRAS* mutation as detected by ddPCR^13^. To confirm the presence of CHCs in peripheral blood specimen prior to subjection to DEP, PBMCs were isolated from whole blood and analyzed as we have previously reported^12,13^. Briefly, PBMCs were adhered to glass slides then stained with antibodies against CD45 and CK, and visualized by fluorescence microscopy (Fig. 3a). The size of CHCs (*n* = 25) from all patients was 9.05 ± 0.89 µm, and was comparable to the size of PBMCs (*n* = 25) 8.57 ± 0.89 µm, confirming the non-significant difference in size between these two populations (Fig. 3b). As indicated, this negates the possibility of size-based isolation as a valid isolation method for CHCs further supporting the utility of label-free DEP enrichment^12^. In addition, these samples had non-detectable levels of CTCs (CD45^−^/CK^+^) by IHC, further supporting the promising nature of CHCs as an important cancer biomarker due to their detectability and potential for harboring clinically relevant information.

**Table 1:** Patient demographics.

Patient ID	Gender	Age	Primary location	Intact primary	Stage at collection	Metastasis	Treatment	1° Tumor *KRAS*^mut^
PDAC3	F	75	Pancreas	yes	IV	lung	yes	p.G12R
PDAC4	F	75	Pancreas	yes	III	no	no	p.G12R
PDAC1	F	53	Pancreas	yes	IV	liver	yes	p.G12D
PDAC2	M	76	Pancreas	yes	III	no	no	p.G12D

Primary (1°) tumor biopsies were clinically tested for *KRAS*.

To demonstrate DEP-enriched CHCs harbored mutant *KRAS*, a smaller aliquot of patient PBMCs were subjected to DEP. A pre-sort aliquot was retained as a control (“Pre-sort”, Fig. 3d). Specimens were loaded onto the device (1 × 10^6^ cells) and all samples were processed for at least 1 h at 15 MHz and 8 Vpp, the optimal conditions for PBMC depletion. Cells were collected from side and center outlets and each channel was subjected to DNA extraction for downstream evaluation of *KRAS* mutations and total DNA was quantified using Qubit^TM^ kits (Fig. 3c). Based on DNA concentration we found that an average of 92.6% ± 2.07 (*n* = 4) of the cells were depleted from the center which is comparable to previous results (Supplementary Fig. 3) but significantly different than healthy controls (*p* = 0.03, unpaired *t* test). PBMCs from cancer patients can be influenced by disease resulting in modified proportions of its subpopulation or by other conditions like immunosenescence where senescent T-cells exhibit abnormal phenotypes^48,49^. These complex factors may influence PBMC DEP response and lead to the utility of such biomarkers. In this way, optimizing the system to deplete healthy PBMCs in a label-free manner may reveal other disease-related cells.

To demonstrate DEP-isolated CHCs harbor detectible *KRAS* mutations, we subjected isolated DNA to ddPCR. We used PBMCs of five screened negative subjects as controls. For all samples, ddPCR probes for the seven most common *KRAS* mutations in PDAC were evaluated. In three out of four patients analyzed, mutant *KRAS* alleles were identified in cells isolated from the center outlet while the side outlets only contained cells expressing wild-type *KRAS* (Fig. 3d). For all other outlets and the screen-negative controls, the number of *KRAS* mutant copies detected were below the LOD for ddPCR^43–46^. PDAC3 had the highest number of *KRAS* mutant copies corresponding to 0.15% of the total DNA, followed by PDAC4 and PDAC1 with 0.13% and 0.1%, respectively. PDAC2 had non-detectable levels of *KRAS* mutations. Although our cohort is limited in number, we identified *KRAS* mutations in 75% of evaluated samples (*p* = 0.048, Fisher Exact test), which is comparable to prior studies that only detected *KRAS*^mut^ CTCs in 72% of samples^50^.

The average *KRAS* mutant copies from the three *KRAS* positive samples was 0.13%. Typically *KRAS* positive cells are heterozygous therefore 0.26% of the cells were positive, corresponding to one *KRAS* mutant cell for every 385 wild-type cells post device processing^51–53^. Previously Dietz et al. found an average of 30 CHCs (*n* = 5) for every 500,000 PBMCs in PDAC patients and found that only 9.1% of CHCs were *KRAS* mutant, therefore the enrichment presented here could be as high as 476-fold. Considering that no CTCs were found by IHC, we believe that *KRAS*^mut^ cells are likely CHCs or other important circulating tumor-derived cells. The level of enrichment presented here is sufficiently above the LOD for current single-cell technologies which could allow for a deeper understanding of circulating cancer cells and associated biology.

## Conclusion

In this study, we present a unique approach addressing the challenge of targeting heterogeneous circulating biomarkers by focusing on healthy PBMCs with defined DEP response to reveal disease-specific characteristics. A DEP-driven microfluidic device fabricated with a V-shape electrode design is ideal for such a strategy. Thus, allowing for continuous mode processing of high cell density samples in a short amount of time. The utility of the workflow was optimized for the depletion of PBMCs permitted maximal enrichment for a CHC model cell line. Using this approach, the device was used to process PBMCs from four PDAC patients and was coupled with genotypic downstream analysis using ddPCR. This workflow allowed for the identification of isolated cells with *KRAS* mutations above the limit of the detection in three out of four patients from only 2 mL of peripheral blood with 1 h of sample processing. We demonstrated DEP-isolated CHCs harbored *KRAS* mutations as a proof of concept, but the overall strategy is applicable to other cancers or diseases with associated cellular biomarkers to provide clinically relevant readouts.

Future work will focus on coupling this technology with droplet-based single-cell technologies where both phenotypic and genotypic information can be studied simultaneously^54,55^. With further biological insights into these potential cellular biomarkers we aim to develop clinical assays beyond *KRAS* mutation status. With the short sample processing time, low input volume, device reproducibility, and compatibility with downstream analytics, our method of cell enrichment by DEP shows promise as a clinical technology with the potential to improve current liquid biopsy strategies of cancer diagnosis and treatment monitoring.

## Materials and methods

### Fabrication and operation of microfluidic DEP sorting device

The microfluidic DEP sorting device described here consists of two layers: a silicon chip with an interdigitated patterned electrode layer and a microfluidic PDMS chamber bonded through plasma treatment and heat curing. The bottom layer consists of three parts, a silicon substrate with 500 nm of wet-growth silicon dioxide (SiO_2_) on both sides, and 90 nm thick of e-beam deposited platinum electrodes as the conductive components for DEP actuation, and silicon dioxide passivated the edge of the electrodes. The electrodes were interdigitated-separated into two batches to form multiple independent electrodes pairs to actuate the sample in the device. The edge of the electrode is designed to be 150 µm from the wall of flow channel, the silicon dioxide passivation avoided cells being trapped at the region where DEP forces are much stronger than the flow stress. The passivation ensures enough shear force to carry sorted cells along the direction of flow.

The device has two DEP actuation region that are connected to two independent pairs of electrode pads. Each electrode pads has two identical circular areas, one was wired bonded by fast drying silver paint (Ted Pella Inc.) and connected to power amplifier (Model 9260, Tabor Electronics Inc) in series with a waveform generator (33622 A, Keysight), the other electrode pad was used to monitor voltage and frequency on the device by connected with an oscilloscope (DSOX2024A, Keysight) at the beginning of each experiment and every time parameters were adjusted. The wired device was placed on a water-cooled aluminum heat sink to keep device at or below room temperature. The complete setup was placed under a microscope for real-time monitoring.

The microfluidic chamber (Supplementary Fig. 1) consists of one sample inlet of 0.5 mm width and two sheath flow inlets of 0.75 mm width for a total ratio of 3:1 sheath to sample ratio. The total chamber width is 2 mm which allowed us to view the entire channel within a field of view by a 5x fluorescence microscope objective as shown in Fig. 2c. At the outlet, to allow only highly responsive cells to be depleted from the sample, we design the center outlet to be wider than the top and bottom outlet. The center outlet is 0.9 mm, and the sides are 0.55 mm each for a total ratio of 11:9 sides to center outlet. The flow is pulled from all three outlets by two flow-driven syringe pumps with a flow rate of 2 µL/min for each of the side outlet and 2.5 µL/min for the center outlet. The withdrawal setup provides enhanced flow stability compared to active infusion from the inlets. The center-focused sample flow at the inlets was adjusted to 5 mm wide by the liquid height of the sheath and sample reservoirs. A common large reservoir for the sheath flow enabled long time stability of the sheath flow and flow confinement of the sample. Both sheath flow and the sample flow were continuously maintained during the cell sorting process (Supplementary Movie 1).

### Microfluidic device preparation and assembly

Polydimethylsiloxane (PDMS, Sylgard 184, Dow Corning, MI) was prepared as the standard 10:1 ratio and cured at 65 °C for at least 2 h and cooled down at room temperature before use. Then, inlets were punctured with 2 mm biopsy puncture (Ted Pella) and outlets were puncture with 1 mm biopsy puncture (Ted Pella). The PDMS part was cleaned by sonication in isopropanol, then PDMS chamber was gas dried followed by incubation in the 80 °C oven for at least 15 min prior to assembly. On the other hand, the electrode patterned silica wafer was sequentially cleaned by acetone, methanol, isopropanol and deionized water. The device was dried by pressurized nitrogen gas prior to assembly.

Both the PDMS microfluidic chamber and silicon chip were treated with oxygen plasma (35 W, 600mtorr) for 1 min. The PDMS chamber was aligned to the fiducial on the silicon chip under a stereoscope and placed on a hot plate at 95 °C for 60 min followed by air cool until room temperature. After PDMS-silicon chip bonding process, 21-gauge wire was wired to the electrode pad using fast drying silver paint (Ted Pella Inc.) and left at room temperature for 1 h before incubated at 80 °C for 1 h to fully dry the silver paint. The 5 min epoxy was applied on the electrode pad and silver paint to enhance the mechanical strength of the wiring. The device was incubated at 80 °C for more 30 min and cooled down to room temperature prior to the experiment.

For each experiment, 20 cm PTFE tubing (24 AWG, Allied Electronics) was connected to a 1 mL syringe. In addition, 0.5 cm tygon tubing (OD: 2.286 mm, ID: 1.270 mm) was plugged into the inlets of the device, a 1 mL pipette was cut approximately in half and inserted on the inlet tubing of the side channels to create a reservoir of sheath flow. A 200 µl pipette was inserted on the tubing on the center to create a sample reservoir. Finally, the device was placed on a recirculating water-cooled aluminum fixture, and monitored by a microscope.

### DEP buffer

To make DEP buffer, 90000 mg of sucrose (Thermo Fisher Scientific), 12086 mg of dextrose (Thermo Fisher Scientific), and 2000 mg of bovine serum albumin (BSA) was dissolved in deionized water (18.2 MOhm-cm) with the total volume of 900 mL. 95 mL of Dulbecco’s modified Eagle’s Media (DMEM) supplemented with 10% fetal bovine serum (FBS) was then added to make a final volume of 950 mL solution. The conductivity of the buffer was adjusted to 145 mS/m by adding DMEM with 10% FBS supplement. The final solution was filtrated by 0.22 µm filter and preserved at 4 °C. This conductivity of the buffer is optimal to provide enough DEP force without causing cells trapped on the electrodes. A lower conductive buffer will cause cell adhering to the electrodes, a higher conductive buffer will require much higher voltage bias.

### Bead sorting

Prior to each experiment DEP buffer was placed under vacuum for at least 30 min to degas it. 10 µm FluoSpheres™ Polystyrene Microspheres and 2.0 µm FluoSpheres™ Carboxylate-Modified Microspheres were diluted to a total of 1 × 10^6^ beads. The bead mixture was then centrifuged and suspended on 100 µl of degassed cold DEP buffer. This solution was then introduced on the microfluidic device and different conditions as described in Fig. 1c were applied. For each condition, the solution was processed for at least 30 min, outlets collected, measured using BD FACS Symphony and analyzed with FlowJo.

### Cell culture

MCF7 (breast adenocarcinoma) were purchased from American Type Culture Collection (ATCC, Manassas, VA) and maintained in Dulbecco’s modified Eagle’s Media (DMEM) supplemented with 10% FBS and 1% Penicillin Streptomycin Glutamine (PSG). B16F10-macrophage hybrids (B16Mϕ) were previously generated and maintained in Dulbecco’s modified Eagle’s Media (DMEM) supplemented with 10% FBS and 1% Penicillin Streptomycin Glutamine (PSG)^12^.

### Cell line PBMC DEP analysis

Prior to each experiment DEP buffer was placed under vacuum for at least 30 min to degas it. Deidentified healthy participant whole blood was obtained from the CEDAR repository following IRB protocol into either a Heparin (BD367874) or ACD (BD364606) vacutainer tubes. Within 15 min of the blood draw, 8–10 mL of whole blood was processed following the SepMate™ (STEMCELL Technologies, cat# 85450) protocol. Cells were counted using the Countess^TM^ II (Applied Biosystems) automated cell counter and diluted to 1 × 10^7^ cells/mL in cold PBS and stored on ice. Cell lines were trypsinized, washed, and reconstituted in DEP buffer. Prior to loading into the DEP device, 100 µl of cell solution was diluted to 1 mL in DEP buffer, centrifuged for 5 min at 300*g*, and reconstituted in 60–80 µl DEP buffer. Reconstituted cells were loaded into the center channel inlet and collected for at least 30 min at each specific frequency and voltage combination. In the case of the spiking experiments, 50,000 cells were added per 1 × 10^6^ PBMCs prior to loading into the device. Each outlet was collected, transferred to standard 5 ml round bottom polystyrene flow cytometry tubes, stained with AF647-CD45(BioLegend, clone H130), PE-CD3 (BioLegend, clone HIT3a), AF488-CD19 (eBioscience, clone HIB19) and V450-CD14 (BD Horizon, clone MφP9) on ice for at least 30 min, measured on BD flow cytometry Symphony, and analyzed with FlowJo. Percentage of depletion and fold of enrichment of a specific subpopulation of PBMCs were calculated using Eq. 3 and Eq. 4 respectively.

3 $${{Depletion}}\,( \% )=\left(1-\frac{\#\,{{of}}\,{X}^{+}\,{{cells}}\,{{in}}\,{{the}}\,{{center}}\,{{outlet}}}{\#\,{{of}}\,{X}^{+}\,{{cells}}\,{{in}}\,{{all}}\,{{three}}\,{{outlets}}}\right)\times 100 \%$$

4 $${{Enrichment}}\,({{fold}})=\frac{\left(\frac{\#\,{{of}}\,{X}^{+}\,{{cells}}\,{{in}}\,{{the}}\,{{center}}\,{{outlet}}}{\#\,{{of}}\,{{total}}\,{{cells}}\,{{in}}\,{{the}}\,{{center}}\,{{outlet}}}\right)}{\left(\frac{\#\,{{of}}\,{X}^{+}\,{{cells}}\,{{in}}\,{{all}}\,{{three}}\,{{outlets}}}{\#\,{{of}}\,{{total}}\,{{cells}}\,{{in}}\,{{all}}\,{{three}}\,{{outlets}}}\right)}$$

### Viability testing

To measure the viability of cells in DEP buffer alone, A549 cells we placed in either DMEM or DEP buffer on ice and serially sampled every 30 min. Each sample was stained with propidium iodide (PI), measured on BD flow cytometry Symphony, and analyzed with FlowJo. PI^+^ cells were considered dead or dying. To measure the viability of cells post-DEP sorting through the device, cells were collected at each outlet and stained with Calcien AM (Invitrogen™ C3099). As before, cells were flow cytometry analyzed and Calcein AM positive cells were considered viable.

### Clinical sample processing for immunofluorescence

PBMCs separation using Ficoll-Paque was describe in^56^ with some modification. 5–10 mL of whole blood was 1: 1 diluted to DPBS with 2% FBS at room temperature. Diluted blood sample was layer on top of 20 mL of Ficoll-Paque PLUS (GE Healthcare), and centrifuged at 800 rcf for 20 min. PBMCs layer was carefully extracted and resuspended in DPBS with 2% FBS. To perform with concentrated cells, the resuspended sample was centrifuged at 400 rcf for 5 min followed by resuspension with intended volume of DPBS with 2% FBS.

For evaluation of CHCs, samples were first treated with a 5% bovine serum albumin solution, followed by TrueBlack Lipofuscin Autofluorescence Quencher (Biotium), and Image-iT FX Signal Enhancer (Invitrogen), then stained with fluorescent-conjugated antibodies for pan-cytokeratin (CK; eBioscience, clone: AE1/AE), and CD45 (Biolegend, clone:HI30), and counterstained with the nuclear dye, DAPI. Each sample was processed with unstained and isotype controls (eBioscience). Specimens were digitally imaged with a Zeiss AxioScanner. PBMC and CHC size was measure using the Zen software ruler.

### Clinical sample processing for DEP device

Deidentified samples were retrieved from Oregon Health & Science University (OHSU) under the Oregon Pancreas Tissue Registry and the Brenden-Colson Center for Pancreatic Care. Informed consent was obtained from all subjects. All experimental protocols were approved by the OHSU Institutional Review Board. All methods were carried out in accordance with relevant guidelines and regulations. At least 1.5 mL of whole blood was processed immediately using SepMate™ (STEMCELL Technologies, cat# 85450) protocol. Cells were counted using Countess^TM^ II (Applied Biosystems) automated cell counter and diluted to 1 × 10^7^cells/mL in cold DEP buffer and stored on ice. As previously described the chip was primed with degassed DEP buffer and the voltage/frequency parameters were set prior to sample loading. The total sample (60–100 µL) was loaded onto the chip and processed for at least 30 min but not more than 60 min.

### DNA extraction and droplet digital PCR

After all outlets were collected samples were centrifuges at 800*g* for 5 min and supernatant was removed. Then Pure™ DNA Extraction Kit was used according to the manufacturer’s protocol. Briefly, 155 µL of digestion buffer was added to the lyophilized proteinase K and resuspended by pipetting up and down for at least 20 times. One fifty microliters of that solution was added to the cell pellets of every outlet and incubated at 65 °C for at least 3 h. After incubation, Ampure XP beads with a 1.2x ratio (v/v) was added and DNA was isolated according to the manufacturer’s protocol. Briefly, samples were under rotation for 10 min prior to magnetic separation of beads and discard of the supernatant. Then 80% ethanol was added to the tube, with samples still in the magnetic rag, and incubated for 30 s before discarding the supernatant. This process was repeated twice and then 11 microliters of water were added to the beads and resuspended. Samples were incubated for 10 min prior to collection of DNA, 10 microliters total, that was either used immediately or store at −20 °C. ddPCR was performed using the ddPCR™ *KRAS* G12/G13 Screening Kit #1863506 (Bio-Rad Laboratories, CA). Droplets were generated with the Auto Droplet Generator (Bio-Rad Laboratories, CA) and measured on the QX200^TM^ Droplet Reader (Bio-Rad Laboratories, CA). PCR parameters were set according to manufacturer recommendations, 95 °C for 10 min, followed by 40 cycles of 94 °C for 30 s and 55 °C for 1 min, followed by 10 min of 98 °C. Mutant and wild-type *KRAS* thresholds were set to ≥ 99% of mutant (A549 cell line) and wild-type (A375 cell line) controls were positive and 100% of blanks were negative.

### Statistics and reproducibility

For Fig. 3c and Supplementary Fig. 3, Unpair t test was performed to determine significance levels using Prism version 9.0 (GraphPad), *P* values are indicated by asterisks; *P* ≤ 0.05 (*) and *P* ≤ 0.0001 (****). For Fig. 3d, Fisher’s exact test was performed to determine significance levels using Prism version 9.0 (GraphPad); *P* = 0.048.

### Reporting summary

Further information on research design is available in the Nature Research Reporting Summary linked to this article.

## Supplementary information

Supplementary Information

Description of Additional Supplementary Files

Supplementary Movie 1

Reporting Summary

## Data Availability

Any remaining information can be obtained from the corresponding author upon reasonable request. Source data for figures are deposited to repository Figshare at: https://figshare.com/projects/Label-free_enrichment_of_rare_unconventional_circulating_neoplastic_cells_using_a_microfluidic_dielectrophoretic_sorting_device/120753
