# Supplementary material for: Label-free enrichment of rare unconventional circulating neoplastic cells using a microfluidic dielectrophoretic sorting device
Source: Commun Biol. 2021 Sep 24;4:1130. doi: 10.1038/s42003-021-02651-8 (PMC8463600; doi:10.1038/s42003-021-02651-8)
Supplement: Supplementary file 1 — Supplementary Information [file 42003_2021_2651_MOESM1_ESM.pdf]

Supplementary Information

Label-free enrichment of rare unconventional circulating neoplastic cells using a microfluidic dielectrophoretic sorting device

Jose Montoya Mira †[1, 2], Ajay A. Sapre †[1], Brett S. Walker[3], Jesus Bueno Alvarez[1], Kyle T. Gustafson[1, 2], Eugene Tu[1], Jared M. Fischer[1, 4, 5], Melissa H. Wong[4, 6], Sadik Esener[1, 2, 4] and Yu-Jui Chiu[1]

[1]Knight Cancer Early Detection Advanced Research Center, Oregon Health & Science University, Portland, OR 97201 USA.

[2]Department of Biomedical Engineering, Oregon Health & Science University, Portland, OR 97201 USA.

[3]Department of Surgery, Oregon Health & Science University, Portland, OR 97239 USA.

[4]The Knight Cancer Institute, Oregon Health & Science University, Portland, OR 97201 USA.

[5]Department of Molecular and Medical Genetics, Oregon Health & Science University, Portland, OR 97239 USA.

[6]Department of Cell, Developmental & Cancer Biology, Oregon Health & Science University, Portland, OR 97201 USA.

Supplementary Information

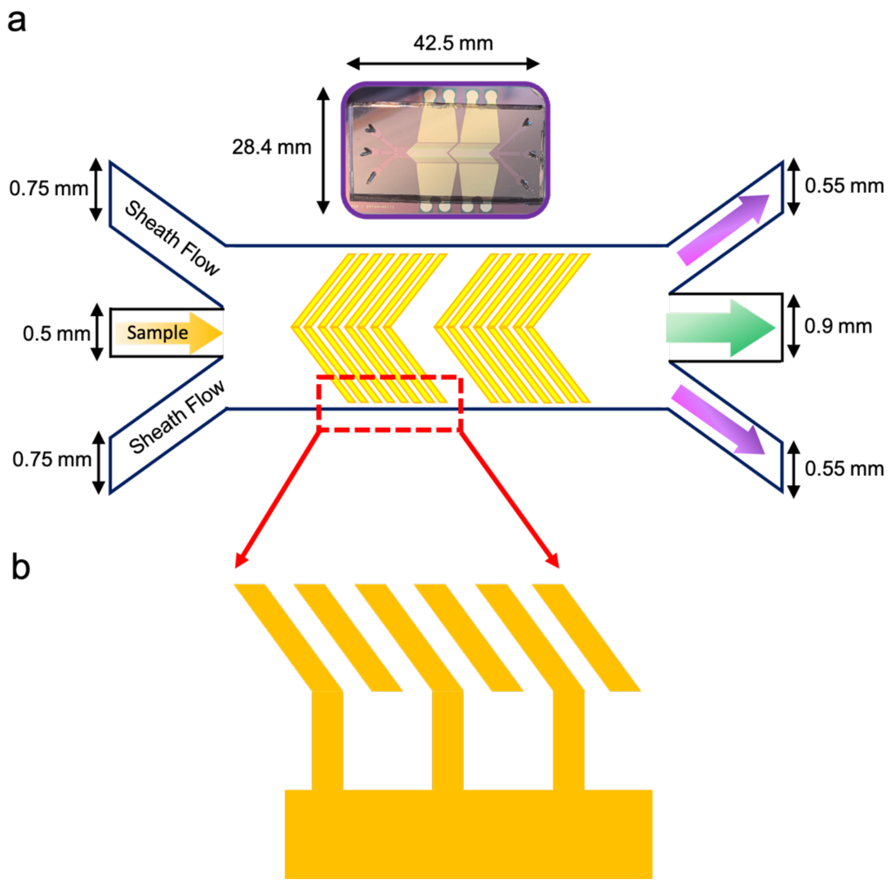

Supplementary Figure 1. (a) Schematic of the microfluidic dielectrophoretic sorting device and real image of the device (top). (b) Eletrode were designed intergiditatedly, half eletrodes were connected to the bottom eletrode pads, the other half were connected to the top eletrode pads.

Supplementary Information

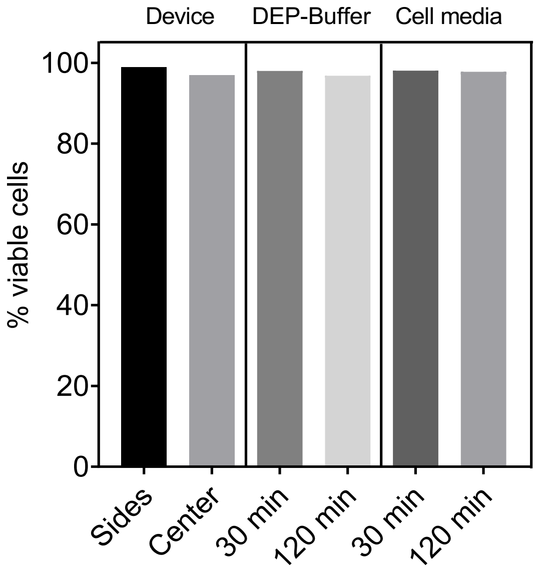

Supplementary Figure 2. Cell viability over incubation time in various buffer conditions. DEP buffer shows high cells viability similar to standard cell culture media.

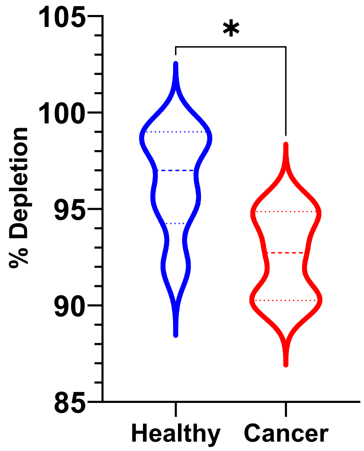

Supplementary Figure 3. Depletion of PBMCs from healthy participants based on Flow cytometry analysis and depletion of PDAC patients PBMCs based on DNA quantification.
