# Supplementary material for: Label-free enrichment of rare unconventional circulating neoplastic cells using a microfluidic dielectrophoretic sorting device
Source: Commun Biol. 2021 Sep 24;4:1130. doi: 10.1038/s42003-021-02651-8 (PMC8463600; doi:10.1038/s42003-021-02651-8)
Supplement: Supplementary file 2 — Description of Additional Supplementary Files [file 42003_2021_2651_MOESM2_ESM.pdf]

Description of Additional Supplementary Files

File name: Supplementary Movie 1.
Description: Peripheral blood mononuclear cells (PBMCs) sorting at 13 MHz with constant voltage of 9 Vpp.
