# Supplementary material for: Label-free enrichment of rare unconventional circulating neoplastic cells using a microfluidic dielectrophoretic sorting device
Source: Commun Biol. 2021 Sep 24;4:1130. doi: 10.1038/s42003-021-02651-8 (PMC8463600; doi:10.1038/s42003-021-02651-8)
Supplement: Supplementary file 4 — Reporting Summary [file 42003_2021_2651_MOESM4_ESM.pdf]

nature portfolio

Corresponding author(s): Yu-Jui Chiu

Last updated by author(s): Aug 18, 2021

# Reporting Summary

Nature Portfolio wishes to improve the reproducibility of the work that we publish. This form provides structure for consistency and transparency in reporting. For further information on Nature Portfolio policies, see our Editorial Policies and the Editorial Policy Checklist.

## Statistics

For all statistical analyses, confirm that the following items are present in the figure legend, table legend, main text, or Methods section.

| n/a | Confirmed | |
|---|---|---|
| ☐ | ☒ | The exact sample size ($n$) for each experimental group/condition, given as a discrete number and unit of measurement |
| ☐ | ☒ | A statement on whether measurements were taken from distinct samples or whether the same sample was measured repeatedly |
| ☐ | ☒ | The statistical test(s) used AND whether they are one- or two-sided<br>*Only common tests should be described solely by name; describe more complex techniques in the Methods section.* |
| ☐ | ☒ | A description of all covariates tested |
| ☒ | ☐ | A description of any assumptions or corrections, such as tests of normality and adjustment for multiple comparisons |
| ☒ | ☐ | A full description of the statistical parameters including central tendency (e.g. means) or other basic estimates (e.g. regression coefficient) AND variation (e.g. standard deviation) or associated estimates of uncertainty (e.g. confidence intervals) |
| ☐ | ☒ | For null hypothesis testing, the test statistic (e.g. $F$, $t$, $r$) with confidence intervals, effect sizes, degrees of freedom and $P$ value noted<br>*Give P values as exact values whenever suitable.* |
| ☒ | ☐ | For Bayesian analysis, information on the choice of priors and Markov chain Monte Carlo settings |
| ☒ | ☐ | For hierarchical and complex designs, identification of the appropriate level for tests and full reporting of outcomes |
| ☒ | ☐ | Estimates of effect sizes (e.g. Cohen's $d$, Pearson's $r$), indicating how they were calculated |

*Our web collection on statistics for biologists contains articles on many of the points above.*

## Software and code

Policy information about availability of computer code

| | |
|---|---|
| Data collection | Zen LITE, BD FACSdiva, QuantaSoft. |
| Data analysis | FlowJo, Prism, BioRad QuantaSoft Analysis Pro. |

For manuscripts utilizing custom algorithms or software that are central to the research but not yet described in published literature, software must be made available to editors and reviewers. We strongly encourage code deposition in a community repository (e.g. GitHub). See the Nature Portfolio guidelines for submitting code & software for further information.

## Data

Policy information about availability of data

All manuscripts must include a data availability statement. This statement should provide the following information, where applicable:

- Accession codes, unique identifiers, or web links for publicly available datasets
- A description of any restrictions on data availability
- For clinical datasets or third party data, please ensure that the statement adheres to our policy

Any remaining information can be obtained from the corresponding author upon reasonable request. Source data for figures are deposited to repository Figshare at: https://figshare.com/projects/Label-free_enrichment_of_rare_unconventional_circulating_neoplastic_cells_using_a_microfluidic_dielectrophoretic_sorting_device/120753

# Field-specific reporting

Please select the one below that is the best fit for your research. If you are not sure, read the appropriate sections before making your selection.

☒ Life sciences ☐ Behavioural & social sciences ☐ Ecological, evolutionary & environmental sciences

For a reference copy of the document with all sections, see nature.com/documents/nr-reporting-summary-flat.pdf

# Life sciences study design

All studies must disclose on these points even when the disclosure is negative.

| | |
|---|---|
| Sample size | Sample size equals four. The sample size here was limited by availability of our repository samples. |
| Data exclusions | No data was excluded from the analysis. |
| Replication | Critical non-clinical experiments were run at least in triplicate if not higher. |
| Randomization | Not applicable. |
| Blinding | Not applicable. |

# Reporting for specific materials, systems and methods

We require information from authors about some types of materials, experimental systems and methods used in many studies. Here, indicate whether each material, system or method listed is relevant to your study. If you are not sure if a list item applies to your research, read the appropriate section before selecting a response.

## Materials & experimental systems

| n/a | Involved in the study | |
|---|---|---|
| ☐ | ☒ | Antibodies |
| ☐ | ☒ | Eukaryotic cell lines |
| ☒ | ☐ | Palaeontology and archaeology |
| ☒ | ☐ | Animals and other organisms |
| ☐ | ☒ | Human research participants |
| ☒ | ☐ | Clinical data |
| ☒ | ☐ | Dual use research of concern |

## Methods

| n/a | Involved in the study | |
|---|---|---|
| ☒ | ☐ | ChIP-seq |
| ☐ | ☒ | Flow cytometry |
| ☒ | ☐ | MRI-based neuroimaging |

## Antibodies

| | |
|---|---|
| Antibodies used | pan-cytokeratin (CK; eBioscience, clone: AE1/AE), CD45 (Biolegend, clone:HI3), AF647-CD45(BioLegend, clone H130), PE-CD3 (BioLegend, clone HIT3a), AF488-CD19 (eBioscience, clone HIB19) and V450-CD14 (BD Horizon, clone MϕP9) |
| Validation | Validated by manufacturer. |

## Eukaryotic cell lines

Policy information about cell lines

| | |
|---|---|
| Cell line source(s) | A549, A375 and MCF7 purchased from ATCC. B16M-RFP generated by C. E. Gast. et al. (DOI: 10.1126/sciadv.aat7828, 2018) |
| Authentication | Authenticated by third party vendor |
| Mycoplasma contamination | Negative by Lonza MycoAlerta Detection Kit |
| Commonly misidentified lines (See ICLAC register) | Not applicable. |

## Human research participants

Policy information about studies involving human research participants

| | |
|---|---|
| Population characteristics | Population information is described in Table 1. |
| Recruitment | *Describe how participants were recruited. Outline any potential self-selection bias or other biases that may be present and how these are likely to impact results.* |
| Ethics oversight | Oregon Health & Science University, OHSU Brenden-Colson Center |

Note that full information on the approval of the study protocol must also be provided in the manuscript.

## Flow Cytometry

### Plots

Confirm that:

- [ ] The axis labels state the marker and fluorochrome used (e.g. CD4-FITC).
- [ ] The axis scales are clearly visible. Include numbers along axes only for bottom left plot of group (a 'group' is an analysis of identical markers).
- [ ] All plots are contour plots with outliers or pseudocolor plots.
- [ ] A numerical value for number of cells or percentage (with statistics) is provided.

### Methodology

| | |
|---|---|
| Sample preparation | PBMCs were obtained from healthy participants via SepMate extraction. They were stained for common known markers such as CD45, CD14, CD19, and CD3. Cell lines were unstained as they expressed wither GFP or RFP/YFP. |
| Instrument | BD FACS Symphony |
| Software | BD FACSdiva and FlowJo |
| Cell population abundance | *Describe the abundance of the relevant cell populations within post-sort fractions, providing details on the purity of the samples and how it was determined.* |
| Gating strategy | Gating was straightforward were CD45+ cells are PBMCs, MCF7 cells are GFP+, and B16CHCs are RFP+/YFP+. For PBMCs unstained controls were used to set thresholds |

- [ ] Tick this box to confirm that a figure exemplifying the gating strategy is provided in the Supplementary Information.
